# HLA-DRB1 and DQB1 Allelic Polymorphism and Multiple Sclerosis in a Moroccan Population

**DOI:** 10.3390/cimb47060458

**Published:** 2025-06-13

**Authors:** Abir Fguirouche, Yahya Naji, Morad Guennouni, Raja Hazime, Safa Zahlane, Mohamed Chraa, Najib Kissani, Nissrine Louhab, Brahim Admou

**Affiliations:** 1 Laboratory of Immunology and HLA, Center of Clinical Research, Mohammed VI University Hospital, Marrakech 40080, Morocco; a.fguirouche.ced@uca.ac.ma (A.F.); ra.hazime@uca.ac.ma (R.H.); 2 Biosciences Research Laboratory, Faculty of Medicine and Pharmacy, Cadi Ayyad University, Marrakech 40080, Morocco; 3 Neurology Department, Faculty of Medicine and Pharmacy, University Hospital of Agadir, Ibn Zohr University, Agadir 80060, Morocco; y.naji@uiz.ac.ma; 4 Science and Technology Team, Higher School of Education and Training, Chouaib Doukkali University, El Jadida 24000, Morocco; m.guennouni@uhp.ac.ma; 5 Neurology Department, Mohammed VI University Hospital, Faculty of Medicine and Pharmacy, Cadi Ayyad University, Marrakech 40080, Morocco; s.zahlan@uca.ac.ma (S.Z.); m.chraa@uca.ma (M.C.); na.kissani@uca.ma (N.K.); ni.louhab@uca.ma (N.L.)

**Keywords:** multiple sclerosis, HLA-DRB1, HLA-DQB1, genetic susceptibility, Moroccan population

## Abstract

**Introduction**: Multiple sclerosis (MS) is a chronic immune-mediated disease of the central nervous system (CNS) that leads to inflammation and demyelination, manifesting in either a relapsing–remitting or progressive form. As a multifactorial disease, MS involves both genetic and environmental factors, with a known significant contribution from human leukocyte antigen (HLA) genes, mainly represented by the HLA-DRB1 and HLA-DQB1 loci, which have been linked to either susceptibility or protection, but variably across populations and ethnic groups. We aimed to study the distribution and polymorphism of HLA-DRB1 and HLA-DQB1 alleles in a population with MS from the southern Moroccan region, in comparison with healthy controls. **Materials and Methods**: A cross-sectional study was conducted over a period of 2 years (2022–2024) in a MS cohort including 40 patients and 100 healthy controls. DRB1 and DQB1 HLA genotyping was performed using a high-resolution reverse sequence-specific oligonucleotide (SSO) method, based on the Luminex system (xMAP technology, One lambda^®^). Data were analyzed using SPSS 26; differences in allele frequencies were evaluated by the Chi-square test and Fisher’s exact test. OR (95% CI) was calculated, and FDR corrections were applied for multiple testing. **Results:** Among the various HLA-DRB1 and DQB1 alleles studied, including those considered as predisposing to MS, the DQB1*02:01 and DRB1*15:01 alleles were more prevalent in MS patients, with 40% and 8.8% vs. 16% and 4.08% in controls respectively, although these differences were not statistically significant (*p* = 0.06 and *p* = 0.12). Likewise, the DRB1*15:01-DQB1*06:02 association was significantly more prevalent in the MS group (9%, *p* = 0.004). In contrast, the DRB1*07:01 allele, linked to protection against MS in many populations, was significantly predominant in controls (17%, *p* = 0.004). Similarly, the DRB1*07:01–DQB*02:01 combination was rather more frequent in controls (12%, *p* = 0.01). Confronted to MS clinical forms, we remarkably noted that the DRB1*13:03 allele was found only among relapsing–remitting MS (RRMS) patients (6%, *p* = 0.003), while DQB1*02:01 was significantly associated with RRMS (42.1%) and primary progressive MS (41%, *p* = 0.001), with an intermediate Expanded Disability Status Scale (EDSS) score, which may indicate a possible link with disease progression and severity. **Conclusions:** The results of our study highlighted particular HLA alleles, DRB1 and DQB1, alone or in combination, as potential immunogenic factors of susceptibility to MS in a population from southern Morocco, while other alleles seem rather to protect against the disease. This HLA polymorphism is also reflected in the clinical forms of the disease, showing a tendency toward severity for certain alleles. However, such preliminary results need to be consolidated and confirmed by studies carried out on a larger population sample, and compared with others on a national scale.

## 1. Introduction

Multiple sclerosis (MS) is a chronic immune-mediated disease of the central nervous system (CNS), marked by inflammatory demyelination and neurodegeneration, resulting in disability among young adults. MS affects approximately 2.8 million individuals worldwide, and many epidemiological and clinical studies have shown the heterogeneity of MS around the world, suggesting a north–south gradient in its incidence influenced by geographic and ethnic origin [1].

MS is considered a complex multifactorial disease arising from an interaction of genetic predisposition and environmental determinants. One of the strongest genetic determinants is the human leukocyte antigen (HLA) class II region, which has been highly associated with MS susceptibility or protection in various populations [2]. Antigenic peptides presented by HLA class II molecules CD4^+^ T lymphocytes are encoded by genes including *HLA-DRB1* and *HLA-DQB1*. The dysregulation of antigen presentation promotes immune tolerance break, leading to autoimmunity and neuro-inflammation in MS [3].

Among HLA class II genes, the HLA-DRB1*15:01 allele is known to be associated with an increased risk of MS in populations of European ancestry, for which studies have shown a relationship between MS susceptibility, early disease onset, and a more aggressive disease course [4,5]. HLA-DRB1*15:01 is believed to enhance the reactivity of T-cells to myelin-derived antigens, resulting in pro-inflammatory immune activation in the CNS, which may impact the severity and progression of the disease [6]. This allele is associated with high levels of pro-inflammatory cytokines, a lower threshold for T-cell activation, and a higher binding affinity to myelin basic proliferating protein (MBP) epitopes, resulting in the initiation of an autoimmune response against the myelin sheath [6].

Other HLA class II alleles have been suggested to act as protective factors, potentially through a mechanism involving the secretion of regulatory T-cell-based responses that inhibit autoimmunity, such as DRB1*11 and DRB1*12 [7].

However, the allelic distribution of HLA-DRB1 and HLA-DQB1 differs in different ethnic groups. In the Middle East and North Africa region (MENA), HLA-DRB1*15:01 has also been identified as a major susceptibility allele, albeit with much lower prevalence than in European populations [1,2,3,4,5,6,7,8]. On the other hand, HLA-DRB1*03, HLA-DRB1*13, and HLA-DQB1*06 have been associated with MS susceptibility among the MENA populations, highlighting the complexity of the genetic background of the disease, which is likely influenced by ethnic and environmental factors [6,7,8,9].

As a part of the MENA region, Morocco is known by its significant genetic diversity due to historical migratory flows and its strategic geographic position [10]. Recent data show an increase in the prevalence of MS in Morocco, with as many as 20 to 40 cases per 100,000 inhabitants [11]. However, genetic studies on MS are still scarce in the country. Thus, understanding the risk and protective alleles for MS may represent a crucial step toward improving disease management and personalized therapeutic strategies for Moroccan patients.

This study aimed to analyze the polymorphism of HLA-DRB1 and HLA-DQB1, and study their possible association with susceptibility or protection from MS among a population from the South of Morocco. It extends our previous investigation of HLA distribution in a healthy Southern Moroccan population linked to MS [12]. Together, these two studies contribute to a broader understanding of the immunogenic background of the Moroccan population linked to MS.

## 2. Materials and Methods

### 2.1. Study Population Selection

A cross-sectional and descriptive study was carried out on 40 patients with MS originally from different areas of the south of Morocco, matched to 100 healthy controls, enrolled over a period of two years (2022–2024), in collaboration with the Neurology Department of the University Hospital. Both recruited patients and controls are characterized by the predominance of Arabs, Amazighs, and Saharans, all originating from the same geographical area including the Marrakech-Safi, Souss-Massa, Guelmim-Oued Noun, and Drâa-Tafilalet governorates

**Patients with MS:** The diagnosis of MS patients was confirmed clinically and radiologically according to the McDonald criteria 2017. This included all patients with MS subtypes: relapsing–remitting MS (RRMS), primary progressive MS (PPMS), and secondary progressive MS (SPMS). Patients who did not meet the criteria mentioned above and those exhibiting other neurological disorders were excluded from this study.

**Control group:** Healthy controls were selected from a pool of potential organ or hematopoietic stem cell donors. These individuals were considered clinically healthy, had no record of neurological diseases, and tested negative for HIV, HBV and HCV.

Sociodemographic and clinical data, including age, gender, geographic origin, disease duration, and Expanded Disability Status Scale (EDSS) score, were recorded for patients with MS, along with relevant past medical history obtained using structured questionnaires for both groups.

### 2.2. Sample Collection

Blood samples were collected according to standardized techniques of venipuncture under aseptic conditions. Participants were asked to offer two 9 mL lithium heparin tubes for HLA class I typing and two 5 mL EDTA (ethylene diamine tetra acetic acid) tubes for HLA class II typing. Nucleated cells were then preserved using the EDTA tubes so that DNA (deoxyribonucleic acid) remained intact for molecular biology analysis.

All samples were placed in transport containers under controlled conditions immediately after collection, then transferred to the HLA Laboratory for further DNA extraction within 24 h of collection.

### 2.3. DNA Extraction and HLA Typing

**DNA extraction:** Genomic DNA was extracted from peripheral blood mononuclear cells (PBMCs) using a QIAmp DNA Mini kit (Qiagen, Hilden, Germany), following a multi-step protocol. Overall, this process began with cell lysis, during which PBMCs were mixed with a lysis buffer containing chaotropic salts. These salts are designed to break membranes and cause the release of genomic DNA, which was then bound onto a silica-based column, enabling DNA purified from other contaminants. Several ethanol-based washing buffers were used during the DNA purification process to remove salts, metabolites, and other compounds. Finally, elution buffer was employed to allow for the release of pure DNA from the membrane without inducing any damage. The quality of isolated DNA was confirmed using a NanoDrop TM 2000/2000c Spectrophotometer (Thermo Scientific TM, Waltham, MA, USA) to dynamically measure concentration and purity, which are essential for downstream molecular analysis.

**HLA Typing:** HLA class II genotyping was performed with reverse sequence-specific oligonucleotide (SSO) DNA typing (Thermo Fisher Scientific, LabType™ XR, Waltham, MA, USA), which is a high-resolution molecular method based on Luminex xMAP technology. This consisted of PCR (polymerase chain reaction) amplification of specific HLA-DRB1 and DQB1 gene regions to enrich the target sequences, and hybridization of PCR-amplified products to probe-coated microspheres under optimized assay conditions. This method allowed for highly specific allele calling, as identified by the fluorescence signals detected from the microspheres via the Luminex system. Each microsphere was tagged with a unique color code for each specific HLA allele probe, so fluorescence intensity was proportional to the amount of hybridization, allowing for accurate allele detection.

Fusion software (version 4.1.0) was used to assess the data, which integrates intensity signals from fluorescence values mapped from Luminex xMAP technology with a known reference database of allele sequences. Deconvolution was facilitated using this software-assisted interpretation for high-dimensionality HLA typing with high-resolution allele calling.

### 2.4. Statistical Analysis

The statistical analysis of clinical and socio-demographic data of the study population (age, age at disease onset, disease duration, and Expanded Disability Scale Score (EDSS)was performed using IBM SPSS 26. The associations of EDSS with HLA class II alleles was assessed using the Kruskall–Wallis test. The comparative analysis of HLA-DRB1 and HLA-DQB1 alleles and haplotype frequencies between MS patients and healthy controls was based on the Chi-square (χ^2^) test or Fisher’s exact test. Two-sided *p*-values were analyzed, and differences were considered statistically significant for *p* ≤ 0.05. In order to analyze the HLA disease association, alleles that strongly deviated from HWE (*p*-value below 0.05) were filtered out using IBM SPSS 26.

We estimated the strength of association of specific HLA alleles/haplotypes with MS susceptibility using odds ratios (ORs) with 95% confidence intervals (CIs).The Benjamini–Hochberg procedure was performed to control the false discovery rate (FDR) correction for multiple testing to provide a conservative threshold for statistical significance.

### 2.5. Ethical Consideration

This study received approval from the Medical Ethics Committee of Mohammed VI University Hospital and Faculty of Medicine and Pharmacy of Marrakech (Ref N°47/2023), ensuring compliance with ethical research standards. Prior to participation, informed consent was obtained from all MS patients. The control group consisted of individuals whose data were collected as part of the routine procedures of the HLA laboratory. Sociodemographic information was retrieved anonymously from medical records for patients and from the HLA laboratory’s database for controls, under the supervision of the respective department heads. Given the nature of the control data collection, individual informed consent was not required.

## 3. Results

### 3.1. Descriptive Analysis of Patients with MS

The mean age of the patients was 40 years ±1.67, which was significantly older than the control group, whose average age was 32 years ±1.88 (*p* = 0.012). In the MS group, a clear female predominance was observed, with 65% females (n = 26) vs. 35% males (n = 14) (Table 1). There was no significant difference in the sex distribution between the two groups (*p* = 0.108). The most frequent clinical subtype of MS observed in our series was relapsing–remitting MS (RRMS), with 47.5% of cases (n = 19), followed by secondary progressive MS (SPMS), with 30% (n = 12), and primary progressive MS (PPMS), which represented 22.5% of cases (n = 9) (Table 1). The mean EDSS score among MS patients was 3.7.

### 3.2. Distribution of HLA-DRB1 Alleles in Patients with MS and Controls

Each locus was tested for Hardy–Weinberg equilibrium (HWE). HLA-DRB1 and HLA-DQB1 genotypes showed no significant deviations from HWE, with *p*-values above the threshold of 0.05 (*p* = 0.911 and *p* = 0.383 for HLA-DRB1 and HLA-DQB1 loci, respectively).

Different HLA-DRB1 allele frequencies were detected among MS patients and healthy individuals. For MS patients, DRB1*07:01 was the most frequent allele, with 10%, followed by DRB1*03:01, with 9.2%, and DRB1*13:03 with 9%, while DRB1*15:02, was the least present in the MS patients, with 5%. The frequency of the other alleles is reported in Table 2. In the control group, the distribution of DRB1 alleles showed a predominance of DRB1*13:01, DRB1*07:01 and DRB1*11:01, with 29%, 17% and 17%, respectively. In return, DRB1*01:01 and DRB1*15:01 were less frequent, with 9% and 4.08%, respectively (Table 2).

A comparative analysis between the two groups showed a statistically significant difference in the distribution of DRB1*07 (*p* = 0.004). For the other alleles, such as DRB1*13:01 and DRB1*11:01, the significant differences observed were *p* = 0.03 and *p* = 0.02, respectively.

### 3.3. Distribution of HLA-DQB1 Alleles in Patients with MS and Controls

Among the main HLA-DQB1 alleles found in our study, DQB1*03:01 was significantly less frequent in MS patients than in controls (12% vs. 30%, *p* = 0.03). Conversely, DQB1*02:01 and DQB1*03:19 were significantly more frequent, with 40% vs. 16%, *p* = 0.06, and 3.7% vs. 0%, *p* = 0.0004. DQB1*06:02, DQB1*05:01, and DQB1*04:01 showed no significant differences between the two groups (Table 3).

### 3.4. Association of HLA DRB1 and DQB1 Alleles in Patients with MS and Controls

As exhibited in Table 4, the analysis of HLA-DRB1 and DQB1 allele associations showed that DRB1*03:01–DQB1*02:01 was the most frequent association, observed in 10.4% of MS patients versus 3% of controls (*p* = 0.01, corrected *p* = 0.49), followed by HLA DRB1*15:01–DQB1*06:02 association, with 9% in patients and 5% in the control group with a statistically significant difference (*p* = 0.004, corrected *p* = 0.009).

Conversely, the DRB1*07:01–DQB1*02:01 association was predominant in healthy controls (13%) as compared with MS patients (1.3%) (*p* = 0.001, corrected *p* = 0.0044). Similarly, DRB1*04:01–DQB1*03:01 was present in 5.0% of MS patients and 7.0% of controls (*p* = 0.45, corrected *p* = 1.00). Lastly, the DRB1*01:01–DQB1*05:01 association was found in 4.0% of patients and 3.05% of controls (*p* = 0.69, corrected *p* = 1.00).

**Figure 1:**
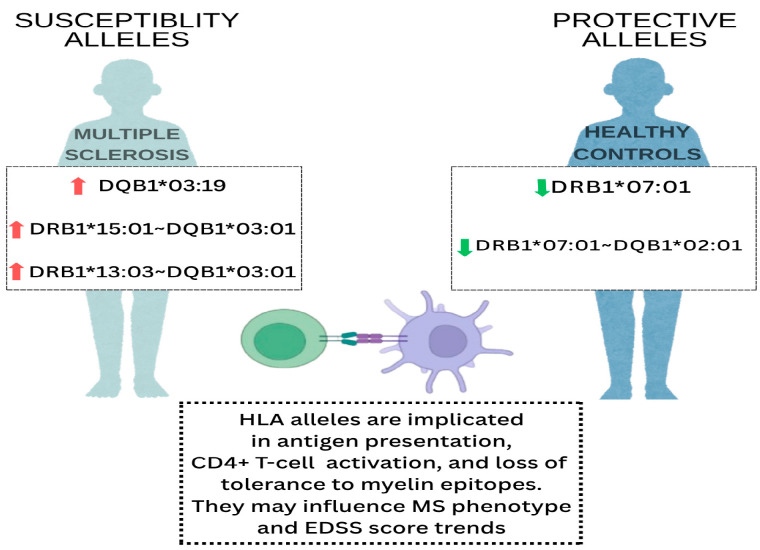
Schematic representation of the main Human Leukocyte Antigen (HLA)class II (DRB1 and DQB1) alleles and haplotypes identified in Moroccan Multiple Sclerosis (MS) patients compared with healthy controls. Upward red arrows indicate alleles more frequent in MS patients, while downward green arrows represent alleles overrepresented in controls.

A schematic representation of the most relevant HLA-DRB1 and HLA-DQB1 alleles and haplotypes associated with susceptibility or protection in our MS cohort is provided in Figure 1.

### 3.5. HLA-DRB1 and DQB1 Allele Polymorphisms and Clinical Forms of MS

#### 3.5.1. Association of DRB1 and DQB1 Alleles with MS Subtypes

The analysis of the HLA-DRB1 and HLA-DQB1 alleles’ distribution according to categories of MS, as illustrated in Table 5, revealed a high predominance of the RRMS subtype among DRB1*03:01 allele carriers (33.6%), followed by DRB1*15:01 (11.1%), then DRB1*13:01 and DRB1*07:01 (10.5% each). SPMS form was detected in 25% of patients displaying the DRB1*03:01 allele, and 16.7% in those with DRB1*04:01. The PPMS category was observed among 31%, 11%, and 10% of patients with DRB1*03:01, DRB1*11:01, and DRB1*07:01 alleles, respectively. We noticed that the DRB1*13:03 allele was seen in a few patients with RRMS patients (*p* = 0.003) and the DRB1*11:01 allele was found only in PPMS patients (0.02).

Regarding the DQB1 locus, we observed that DQB1*02:01 allele was equally predominant in RRMS patients (42.1%) and SPMS (41%), with a significant association (*p* = 0.001). Similarly, DQB1*03:01 was significantly prevalent in RRMS patients, with 44.5% (*p* = 0.009).

#### 3.5.2. DRB1 and DQB1 Allele Polymorphisms and EDSS Score in MS

By matching the HLA-DRB1 and DQB1 alleles with the Expanded Disability Scale Status (EDSS) score of the disease, indicating the severity degree of disability during MS course, we noticed that DQB1*06:02, DRB1*15:01, DRB1*13:01, and alleles were associated with higher disability trends, with the mean EDSS scores estimated at 6, 5.8, and 5 respectively. Conversely, DRB1*11:01, DRB1*04:01, and DQB1*05:01 were linked to lower disability trends, with EDSS mean scores inferior to 3 points. Other alleles, like DRB1*07:01, DQB1*03:01, and DQB1*02:01, (EDSS = 3.5) and (EDSS = 4), displayed intermediate disability trends, with EDSS mean scores varying between 3 and 4. However, no statistically significant difference was observed in EDSS scores within different alleles (Table 6).

## 4. Discussion

Our study examined HLA class II alleles; notably, DRB1 and DQB1, some of which are known for their potential association with MS. The population studied originated from different areas of Southern Morocco, characterized by significant ethnic diversity combining Arabs, Berbers, and Saharans.

The female predominance observed in our series (sex-ratio = 1:2) aligns with data reported in the literature. Indeed, the prevalence of MS presents a gender disparity, but more in favor of women than men. This gender difference is thought to be linked to the impact of estrogen and progesterone on inflammatory pathways, especially through the modulation of T-cell activity [13,14].

Evidence for genetic involvement during the course of MS has been highlighted in different ancestral groups. In monozygotic twins, the concordance risk is around 30% compared with approximately 3% in dizygotic twins [6]. These studies have demonstrated the significant implication of HLA class II alleles in disease progression [1]. The results of our study revealed no association of DRB1 and DQB1 alleles with age at disease onset (33 years old). Some authors have rejected such an association [8,9,10,11,12,13,14,15], while [16] confirmed this statement only for elderly. Moreover, the allele frequencies of both DRB1 and DQB1 loci are in HWE and may represent a well-established population with no strong selective pressure on these loci in relation to MS.

The well-established relationship between DRB1*15:01 and MS risk has been steadily reported among different ethnicities. Notably, Caucasians [6], Latin Americans [17], Chinese [18], and North Africans [9]. Using four-digit high-resolution genotyping, our findings highlighted the presence of DRB1*15:01 among 8.8% of the Moroccan MS population vs. 4.08% in healthy controls (*p* = 0.12), while the risk of developing MS was 2.18. Similarly, Ouadghiri et al. [19] reported OR = 2.67 in a Northern Moroccan population (Rabat), yet conversely, with a significant association with DRB1*15:01 to MS. A potential geographic and genetic heterogeneity could explain this regional disparity.

The genetic pool in North African populations has been influenced by major historical migration events, including Arabs, Berbers, Phoenicians, and Saharans, which may be responsible for reducing the prevalence of DRB1*15:01 compared with Europeans [10]. Other susceptibility alleles, like DRB1*03:01, DRB1*13:01, and DRB1*04:01, were noted among our MS patients with 9.2%, 9%, and 7%, respectively, with a potential trend toward association with MS susceptibility, especially for DRB1*13:01. Molecular analysis studies showed the important implication of these alleles during autoantigen presentation to T lymphocytes due to their shared amino acids [6]. In the MENA region, DRB1*04 and DRB*03 highlighted a predisposing role for MS [1]. Moreover, studies from Sardinia and Turkey reported the implication of DRB1*04 in MS susceptibility [1], while DRB1*03 was linked to MS predisposition in Portugal [20], the United Kingdom [19], and Sweden [21]. In many populations with different ethnicities, mainly Arabs, Canadians, and Brazilians, alleles like DRB1*07, DRB1*11, and DRB1*01 were linked with reducing MS susceptibility [1,2,3,4,5,6]. Interestingly, in our studied population, DRB1*07:01 was significantly predominant in the control group with 17% compared with MS patients (*p* = 0.004), suggesting a protective role. Additionally, DRB1*11:01 and DRB1*01:01 expressed significant associations with protection against MS, with *p* = 0.03 and *p* = 0.05, respectively. These findings are consistent with our previous study conducted on a healthy population from Southern Morocco [12]. The underlying mechanism of protection remains unclear, yet many studies suggest the modulation of regulatory T-cell function and antigen efficiency as potential protective mechanisms [22].

The HLA-DQB1 polymorphism was also explored, since it is known to play a complementary role with HLA-DRB1 in MS pathogenesis. Our results showed the presence of the DQB1*02:01 in 40% of MS patients (*p* = 0.06), which aligns with results reported by Aljumah et al. [8]. Similarly, DQB1*06:02 is known to raise the risk of MS among Caucasians and Europeans [23], was seen in 10% of MS patients. Furthermore, our study identified DQB1*03:19 exclusively in a few MS patients (3.7%), and in none of the controls. This led to formally infinite OR, due to the complete absence of the allele in controls. Although statistically significant (*p* = 0.0004), this finding requires caution and further validation in larger cohorts, as it may suggest a potentially novel risk allele within Moroccan MS patients.

Using the high-resolution four-digit genotyping technique, our findings confirmed the crucial role of DRB1*15:01–DQB1*06:02 as an important risk combination (*p* = 0.004), enhancing its global importance as a risk factor for MS. This result was reported in many populations with different genetic backgrounds [6]. It has been identified consistently among Europeans [4], North Americans [5], and North Africans [9,10,11,12,13,14,15,16,17,18,19]. Additionally, DRB*13:03-DQB1*03:01 was present in 6.2% of the MS population while being absent among healthy controls (*p* = 0.00038). Studies using high-resolution genotyping techniques in the MENA region are scarce; thus, inconsistencies have been reported regarding DQB1A frequency during MS course [1]. In contrast, the DRB1*07:01–DQB1*02:01 combination was significantly more frequent in controls (13%) compared with MS patients (1.3%) (*p* = 0.001). This supports its protective role against MS.

Furthermore, we analyzed the association of HLA alleles with MS subtypes (RRMS, SPMS, and PPMS) and EDSS score to assess their influence on MS progression, which showed that HLA–DRB1*03:01 was the most frequent allele in RRMS patients (33.6%) with moderate disability (EDSS = 3.4), followed by DRB1*15:01, seen in 11% among the same group with higher disability trend (EDSS = 5); although these alleles appeared more common in certain subtypes or EDSS ranges, these trends were not statistically significant and must be interpreted as exploratory until validated in larger, independent cohorts. Some studies previously linked severe disease course and a high EDSS score [24]. Other alleles, such as DRB1*13:03, were found only among one RRMS patient (*p* = 0.003), suggesting potential immune-mediated relapsing activity. Among the HLA DQB1 alleles, DQB1*02:01 was significantly associated with RRMS (42.1%) and SPMS (41%) (*p* = 0.001), with an intermediate EDSS score. These results suggest that HLA class II alleles may contribute differently to MS progression. Some of them may predispose to relapsing–remitting forms, while others may be linked to progressive neurodegeneration, underscoring the need for larger scale studies to confirm these possible trend associations.

These findings support the hypothesis that, in the context of MS, HLA DR and DQ alleles influence antigen presentation and immune response modulation by favoring the polarization of CD4+ T helper cells toward TH1 and TH17 responses [25]. Additional genetic, environmental, and other factors, such as vitamin D deficiency, smoking, and Epstein–Barr Virus infection, are potentially associated with MS course and severity [8].

The distinctive HLA DR and DQ allele distribution observed in our population is certainly influenced by centuries of migration and admixture among Berbers, Arabs, and Saharans as part of the genetic diversity of Morocco [10].

Despite the originality and the value of our study, we acknowledge several limitations, notably the relatively small sample size for the detection of rare allele associations. Therefore, the possibility of type II errors cannot be excluded and some true associations may have gone undetected. Furthermore, the focus was on a single region, which may not reflect the full heterogeneity of the Moroccan population.

## 5. Conclusions

The data of the current study highlighted a high frequency of DRB1*03:01, DRB1*15:01, DRB1*04:01, and DQB1*03:19 HLA alleles, along with DRB1*15:01-DQB1*06:02 association, which are linked with a possible trend of association with the risk of MS within the population of southern Morocco. In contrast, the DRB1*07:01 allele and DRB1*07:01–DQB1*02:01 combination may be considered as protective immunogenic factors from MS.

Certain alleles appeared more frequently in specific MS forms, either relapsing–remitting or primary progressive forms, suggesting possible trend associations of these alleles with MS progression and severity that require further confirmation.

However, these preliminary results need to be consolidated by studies carried out on a larger population sample and compared with others on a national scale.

Our results provide promising insights into the etiopathogenic determinism of MS, which can serve as a basis for personalized therapeutic strategies based on patients’ immunogenic profiles, the identification of which could greatly facilitate early diagnosis and better management of the MS population.

## Figures and Tables

**Table 1:** Demographic and clinical characteristics of MSpatients.

Demographic Characteristics	MS Patients	Controls	*p*-Value
**Mean age (years)**	40 ± 1.67	32 ± 1.88	0.012
**Male (%)**	35%	50%	0.108
**Female (%)**	65%	50%	0.108
**Clinical subtypes**		**Nb of patients (n)**	**Percentage (%)**
**Relapsing–Remitting MS (RRMS)**		19	47.5%
**Secondary Progressive MS (SPMS)**		12	30.0%
**Primary Progressive MS (PPMS)**		9	22.5%

**Table 2:** Comparison of DRB1 allele frequencies between MS patients and controls.

HLA DRB1 Alleles	Frequency in Patients, n = 40 (%)	Frequency in Controls, n = 100 (%)	Odds Ratio	95% CI	*p*-Value	c-p*
DRB1*15:01	8.8	4.08	2.18	0.79–6.04	0.12	0.36
DRB1*15:02	5	2	2.58	0.35–18.97	0.32	1
DRB1*03:01	9.2	8.43	1.15	0.61–2.14	0.67	0.89
DRB1*13:01	4	29	0.13	0.03–0.57	0.03	0.25
DRB1*13:03	9	14.5	0.32	0.07–1.49	0.15	1
DRB1*07:01	**10**	**17**	**0.41**	**0.14–1.38**	**0.004**	**0.027**
DRB1*04:01	7.0	5.0	0.69	0.22–2.14	0.45	1
DRB1*11:01	1	17	0.12	0.03–1.00	0.03	0.25
DRB1*01:01	1	9	0.0	0.01–2.09	0.05	0.34

c-p*: Corrected *p*-value with false discovery rate (FDR) test. Alleles with statistical significance after FDR correction are indicated in bold. 95% CI: Confidence Interval.

**Table 3:** Comparative frequencies of HLA-DQB1 alleles between patients and controls.

HLA DQB1 Alleles	Frequency in Patients, n = 40 (%)	Frequency in Controls, n = 100 (%)	Odds Ratio	95% CI	*p*-Value	c-p*
DQB1*06:02	10	6	1.74	0.47–6.41	0.10	0.30
DQB1*02:01	40	16	0.48	0.23–1.02	0.06	0.39
DQB1*03:19	**3.7**	**0**	**∞**	**0.22–∞**	**0.0004**	**0.0011**
DQB1*03:01	12	30	0.42	0.20–0.95	0.03	0.11
DQB1*05:01	4.0	3	1.33	0.36–4.90	0.72	0.69
DQB1*04:01	2.0	1.5	1.35	0.31–5.88	0.49	0.56

∞ means that the allele was not seen in any of the control group. c-p*: Corrected *p*-value with false discovery rate (FDR) test. Alleles with statistical significance after FDR correction are indicated in bold. 95% CI: Confidence Interval.

**Table 4:** Comparative analysis of associated HLA DR–DQ allele frequencies in patients and controls.

HLA DR–DQ Associations	Frequency in Patients (%)	Frequency in Controls (%)	Odds Ratio	95% CI	*p*-Value	c-p*
DRB1*15:01–DQB1*06:02	**9**	**5**	**1.8**	**1.5–5.4**	**0.004**	**0.009**
DRB1*13:03–DQB1*03:01	**6.2**	**0.0**	**∞**	**1.42–∞**	**0.0004**	**0.0011**
DRB1*07:01–DQB1*02:01	**1.3**	**13**	**0.10**	**0.02–0.42**	**0.001**	**0.044**
DRB1*03:01–DQB1*02:01	10.4	3	0.11	0.04–0.85	0.01	0.49
DRB1*04:01–DQB1*03:01	5.0	7.0	0.69	0.22–2.14	0.45	1
DRB1*01:01–DQB1*05:01	4.0	3.05	1.33	0.36–4.90	0.69	1

∞ means that the allele/haplotype was not seen in any of the control group. c-p*: Corrected *p*-value with false discovery rate (FDR) test. Alleles with statistical significance after FDR correction are indicated in bold. 95% CI: Confidence Interval.

**Table 5:** Association of DRB1 and DQB1 alleles with clinical forms of MS.

DR and DQ Alleles	PPMS %	RRMS %	SPMS %	*p*-Value *
DRB1*15:01	3	11.1	8.3	0.50
DRB1*03:01	31	33.6	25	0.66
DRB1*13:01	0	10.5	8.3	0.66
DRB1*13:03	**0**	**6**	**0**	**0.003**
DRB1*07:01	10	10.5	8.3	0.83
DRB1*04:01	0	5.3	16.7	0.48
DRB1*11:01	**11**	**0**	**0**	**0.02**
DQB1*02:01	**2**	**42.1**	**41.7**	**0.001**
DQB1*06:02	1	10.5	25	0.71
DQB1*03:19	0	5	2.5	0.15
DQB1*03:01	**10.5**	**44.5**	**8.3**	**0.009**
DQB1*05:01	0	5.3	8.3	0.76
DQB1*04:01	0	8.3	0	0.06

* Pearson Khi-square test was used for MS subtypes associations. PPMS: Primary Progressive Multiple Sclerosis, RRMS: Relapsing-remetting Multiple Sclerosis, SPMS: Secondary Progressive Multiple Sclerosis.

**Table 6:** EDSS score according to DRB1 and DQB1 alleles.

DR and DQ Alleles	Mean EDSS Score	Trend	*p*-Value *
DRB1*15:01	5	Higher disability	0.548
DRB1*03:01	3.4	Moderate disability	0.548
DRB1*13:01	5.8	Higher disability	0.548
DRB1*13:03	4	Moderate disability	0.548
DRB1*07:01	4.4	Moderate disability	0.548
DRB1*04:01	2.5	Low disability	0.548
DRB1*11:01	2	Moderate disability	0.548
DQB1*02:01	3.5	Moderate disability	0.763
DQB1*06:02	6	Higher disability	0.763
DQB1*03:19	4.3	Moderate disability	0.763
DQB1*03:01	4	Moderate disability	0.763
DQB1*05:01	3	Moderate disability	0.763
DQB1*04:01	3	Moderate disability	0.763

*p*-value * obtained using Kruskall–Wallis for Expanded Disability Scale Status (EDSS) associations.

## Data Availability

The data in this study are not publicly available due to privacy restrictions. Researchers interested in accessing the data for replication or verification may request it through the institutional review board, subject to approval and privacy compliance. For data access inquiries, please contact Pr. Brahim ADMOU at the Clinical Research Center, Mohammed VI University Hospital Center (email: br.admou@uca.ac.ma).

## Abbreviations

The following abbreviations are used in this manuscript:

MS	Multiple Sclerosis
HLA	Human Leukocyte Antigen
CNS	Central Nervous System
SSO	Sequence-Specific Oligonucleotide
MBP	Myelin Basic Protein
RRMS	Relapsing–Remitting Multiple Sclerosis
PPMS	Primary Progressive Multiple Sclerosis
SPMS	Secondary Progressive Multiple Sclerosis
EDTA	Ethylene Diamine Tetraacetic Acid
DNA	Deoxyribonucleic Acid
PCR	Polymerase Chain Reaction
PBMCs	Peripheral Blood Mononuclear Cells
ORs	Odds Ratios
CIs	Confidence Intervals
FDR	False Discovery Rate
EDSS	Expanded Disability Status Scale
MENA	Middle East and North Africa
